# Effects of reactive social distancing on the 1918 influenza pandemic

**DOI:** 10.1371/journal.pone.0180545

**Published:** 2017-07-12

**Authors:** Duo Yu, Qianying Lin, Alice PY Chiu, Daihai He

**Affiliations:** 1 Department of Applied Mathematics, Hong Kong Polytechnic University, Hung Hom, Kowloon, Hong Kong (SAR), China; 2 Department of Biostatistics, School of Public Health, University of Texas, Health Science Center at Houston, Houston, United States of America; Georgia State University, UNITED STATES

## Abstract

The 1918 influenza pandemic was characterized by multiple epidemic waves. We investigated reactive social distancing, a form of behavioral response where individuals avoid potentially infectious contacts in response to available information on an ongoing epidemic or pandemic. We modelled its effects on the three influenza waves in the United Kingdom. In previous studies, human behavioral response was modelled by a Power function of the proportion of recent influenza mortality in a population, and by a Hill function, which is a function of the number of recent influenza mortality. Using a simple epidemic model with a Power function and one common set of parameters, we provided a good model fit for the observed multiple epidemic waves in London boroughs, Birmingham and Liverpool. We further applied the model parameters from these three cities to all 334 administrative units in England and Wales and including the population sizes of individual administrative units. We computed the Pearson’s correlation between the observed and simulated for each administrative unit. We found a median correlation of 0.636, indicating that our model predictions are performing reasonably well. Our modelling approach is an improvement from previous studies where separate models are fitted to each city. With the reduced number of model parameters used, we achieved computational efficiency gain without over-fitting the model. We also showed the importance of reactive behavioral distancing as a potential non-pharmaceutical intervention during an influenza pandemic. Our work has both scientific and public health significance.

## Introduction

The influenza pandemic of 1918 had been regarded as the deadliest pandemic in history. It had caused an estimated 50 to 100 million deaths worldwide [1, 2]. Due to its exceptional lethality and unusual epidemiological features, an in-depth understanding of the 1918 pandemic could provide insights to future influenza pandemic control and intervention. The 1918 pandemic was characterized by multiple waves of mortality. In the United Kingdom, the pandemic took place as three distinct waves: the first wave in the summer 1918, the second wave in the autumn of the same year, and the third wave in the spring of 1919.

Behavioral epidemiology of infectious diseases focuses on behavior of the individuals as a key factor to predict infection trajectories [3]. “Reactive social distancing” is a form of behavioral responses where individuals avoid potentially infectious contacts in response to available information on an ongoing epidemic or pandemic. Such behavioral responses could include avoiding mass gathering, putting on protective masks, actively maintaining personal hygiene and getting vaccinated [4]. However, the exact nature of behavioral responses could vary from disease to disease.

Many previous studies investigated multiple waves of 1918 influenza pandemic. Merler et al. proposed co-infections as a determinant of multiple waves [5]. Several other studies have focused on identifying the underlying causes of multiple waves and the impact of behavioral responses [6–8]. He et al. showed that human behavioral response was a key factor that was responsible for the temporal changes in transmission rates of the three epidemic waves in England and Wales. They described the behavioral response in the form of a Power function which described the proportion of recent mortality over the whole population, and said response has the largest impact on the epidemic waves of weekly infections [6]. Poletti et al. studied the 2009 H1N1 influenza pandemic and concluded that human behavioral changes responding to the total number of infections could have a significant impact on the timing, dynamics and magnitude of the epidemic spread [7]. Bootsma and Ferguson modelled the behavioral responses in the form of the Hill function which described the number of mortality. They showed that the response could have a stronger impact on the weekly deaths than on the overall mortality [8]. These studies all pointed towards human behavioral responses being a key factor in the occurrence of multiple waves in the 1918 influenza pandemic.

Although previous studies investigated the impact of behavioral responses on the 1918 influenza pandemic, many questions remain unanswered. The impact of reactive social distancing on the final epidemic size remains unknown. Previous studies which used an epidemic model that included a common set of parameters for different cities did not result in a good model fit. They also lacked a comparison of different forms of functions describing behavioral responses [6, 8]. In this study, we aimed to compare two mathematical functions of reactive social distancing: Power function, which is a function of the proportion of recent influenza mortality in a population, and Hill function, which is a function of recent influenza mortality.

This manuscript is arranged as follows: we fitted our model with the same set of input parameters to the observed data in London boroughs, Birmingham and Liverpool. We applied those model parameter values obtained from fitting three cities to all 334 administrative units in England and Wales using population sizes of individual administrative units. We estimated the impact of reactive social distancing on the final epidemic size. In the S1 Appendix, we demonstrated theoretically how oscillations are induced by reactive social distancing.

## Methods

### Data

We analysed data on weekly influenza deaths between June 29, 1918 and May 10, 1919 from 334 administrative units in London boroughs, Birmingham and Liverpool in England. These data are publicly available from UK Data Services [9].

Daily temperature data for the same time period are obtained from UK Met Office Hadley Centre for Climate Change, where they provided Central England temperature data (http://www.metoffice.gov.uk/hadobs/hadcet/data/download.html).

### Social distancing

Behavioral functions are used to model how people reduce their exposure to potentially infectious contacts in response to the reported mortality during an influenza pandemic [6, 8]. In Eq (1), we described three forms of behavioral functions (*B*(*W*(*t*)) that could affect the transmission rates: the Power function [6], the Hill function [8] and the modified-Hill function. We denote the recent mortality as *W*(*t*), the total population size as N, and the intensity of behavioral response as *κ* or *N*/*κ*.

We compared the Taylor series expansion of the Power function and the Hill function at *W*(0) = 0. We noted that the coefficient of *W*(*t*) in the Hill function, *N*/*κ*, is comparable to the *κ* in the Power function. They both indicated the intensity of behavioral responses which depended on the perceived risk of infection. Furthermore, we obtained a Modified-Hill function when we replaced *κ* with *N*/*κ* in the Hill function. The first two terms of the Taylor series expansion in the Power function and the modified-Hill function are identical.

$$\begin{array}{ccc} & & B(W(t);\kappa ,N)=\\ & & \;\text{Power}\;\text{function}\;{\left[1-\frac{W(t)}{N}\right]}^{\kappa }=1-\kappa \frac{W(t)}{N}+\frac{\kappa (\kappa -1)}{2}{\left[\frac{W(t)}{N}\right]}^{2}+\cdots \end{array}$$ (1a)

$$\begin{array}{ccc} & & \text{Hill}\;\text{function}\;\frac{\kappa }{\kappa +W(t)}=1-\frac{1}{\kappa }W\left(t\right)+\frac{1}{\kappa^{2}}W{\left(t\right)}^{2}+\cdots \end{array}$$ (1b)

$$\begin{array}{ccc} & & \text{Modified-Hill}\;\text{function}\;\frac{1/\kappa }{1/\kappa +W(t)/N}=1-\kappa \frac{W(t)}{N}+\kappa^{2}{\left[\frac{W(t)}{N}\right]}^{2}+\cdots \end{array}$$ (1c)

**Fig 1:**
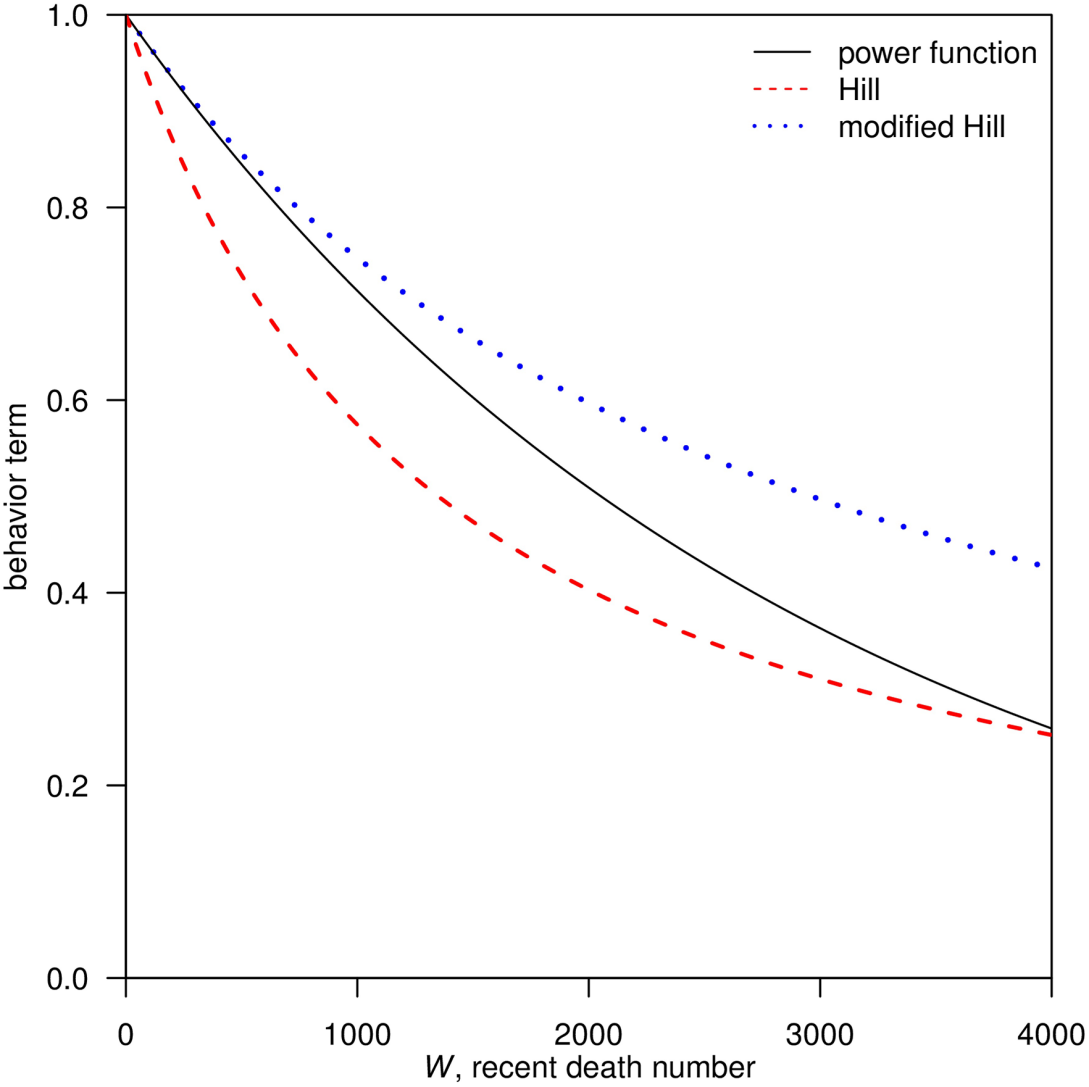
Simulation comparison of the three behavioral functions using the same parameter settings: *N* = 4000,000, *κ* = 1350. Black line, red dashed line and blue dotted line represent Power function, Hill function and modified-Hill function, respectively.

As shown in Fig 1, when $\frac{W(t)}{N}$ is close to zero, the Taylor series expansion suggests that the Power function and the modified-Hill function lead to almost the same value. However, when the proportion of recent mortality gets larger, the value of the Power function approaches those of the Hill function. The key difference is whether *W*(*t*) is scaled by *N* or not, i.e. whether we are considering the proportion of recent mortality or the exact mortality.

### Model

We employed a simple Susceptible-Infectious-Recovered (SIR) model which included a behavioral function. Similar results were achieved if an additional “exposed” class is included [6]. The model is represented as follows:

$$\begin{array}{ccc} \dot{S} & = & -\frac{\beta (t)}{N}SI,\\ \dot{I} & = & \frac{\beta (t)}{N}SI-\gamma I,\\ \dot{R} & = & \gamma \cdot (1-\phi )I,\\ \dot{D} & = & \gamma \phi I-gD,\\ \dot{M} & = & gD,\\ \dot{W} & = & gD-\lambda W. \end{array}$$ (2)

As in Eq (2), we assumed people behaved with reactive social distancing according to *W*, where such behavior could be described as an exponential function with decaying rate *λ*, which referred to the exponentially fading memories of information variables in behavioral epidemiology [4]. As in previous studies on the 1918 pandemic, we adopted the simplifying assumption that population size (*N*) is constant throughout the pandemic course, although this assumption is not realistic due to the large number of influenza mortality. The population sizes in London boroughs, Birmingham and Liverpool were approximately 4,484,523, 919,444 and 802,940 during the study period respectively. *ϕ* denotes the case-fatality ratio. Parameters *γ*, *g* and *λ* are rates at which individuals moved from one class to the next. *γ*^−1^ is the mean infectious period, fixed at four days [10]. *g*^−1^ is the mean time from loss-of-infectiousness to death, fixed at eight days. Thus, the mean duration from infection to death is 12 days [8]. *λ*^−1^ is the mean duration of delay in behavioral responses. Following [6], *β*(*t*) is the transmission rate function which takes the following form:

$$\begin{array}{c} \beta \left(t\right)=\beta_{0}\cdot e^{-\xi T(t)}\cdot \left[1+\alpha H\left(t\right)\right]\cdot B\left(W\left(t\right);\kappa ,N\right), \end{array}$$ (3)

where the four components are described as follows:

1. *β*_0_ is the constant baseline transmission rate.
2. *e*^−*ξT*(*t*)^ is the term representing the temperature effect, *T*(*t*) is the daily time series for temperature, where we simulated from the weekly time series obtained from UK Met Office. The parameter *ξ* describes the intensity of the temperature effect.
3. [1 + *αH*(*t*)] is the school term factor. It has an amplitude parameter *α* and a school day function *H*(*t*). *H*(*t*) is a step function that takes a large value on school days and a small value on holidays [6]. Easter and Christmas holidays are known. The summer vacation period (*t*_1_, *t*_2_), which are unknown, has to be estimated. During the 1910’s, a large number of adults and school children were involved in summer harvesting. Thus there were an impact on influenza transmission [6].
4. The last factor is the human behavioral term *B*(*W*(*t*);*κ*, *N*) that is expressed differently for the Power function, Hill function and modified-Hill function. *W*(*t*) denotes the recent influenza mortality. *κ* or *N*/*κ* represent the intensity of human behavioral response towards the perceived risk of influenza infection.

Finally, we define the basic reproductive number $R_{0}=\frac{\langle \beta (t)\rangle }{\gamma }$ with *W*(*t*) = 0. We have *W* = 0 at disease-free equilibrium [6]. Since the temperature effect is on average smaller than one, we have 〈*β*(*t*)〉 < *β*_0_. However, we chose to estimate the effective reproductive number, $R_{\text{eff}}\left(t\right)$, instead. $R_{\text{eff}}\left(t\right)$ is defined as the average number of secondary case of infection per primary case at time *t* [11]. It is more appropriate to use $R_{\text{eff}}\left(t\right)$ than *R*_0_ in this study because we need to estimate the final epidemic size, which reflects the depletion of susceptible individuals under reactive social distancing. We have $R_{\text{eff}}\left(t\right)=\beta \left(t\right)S\left(t\right)/\left(\gamma N\right)$ [12]. An $R_{\text{eff}}\left(t\right)\leq 1$ indicates that the epidemic is under control.

### Modelling framework

**Fig 2:**
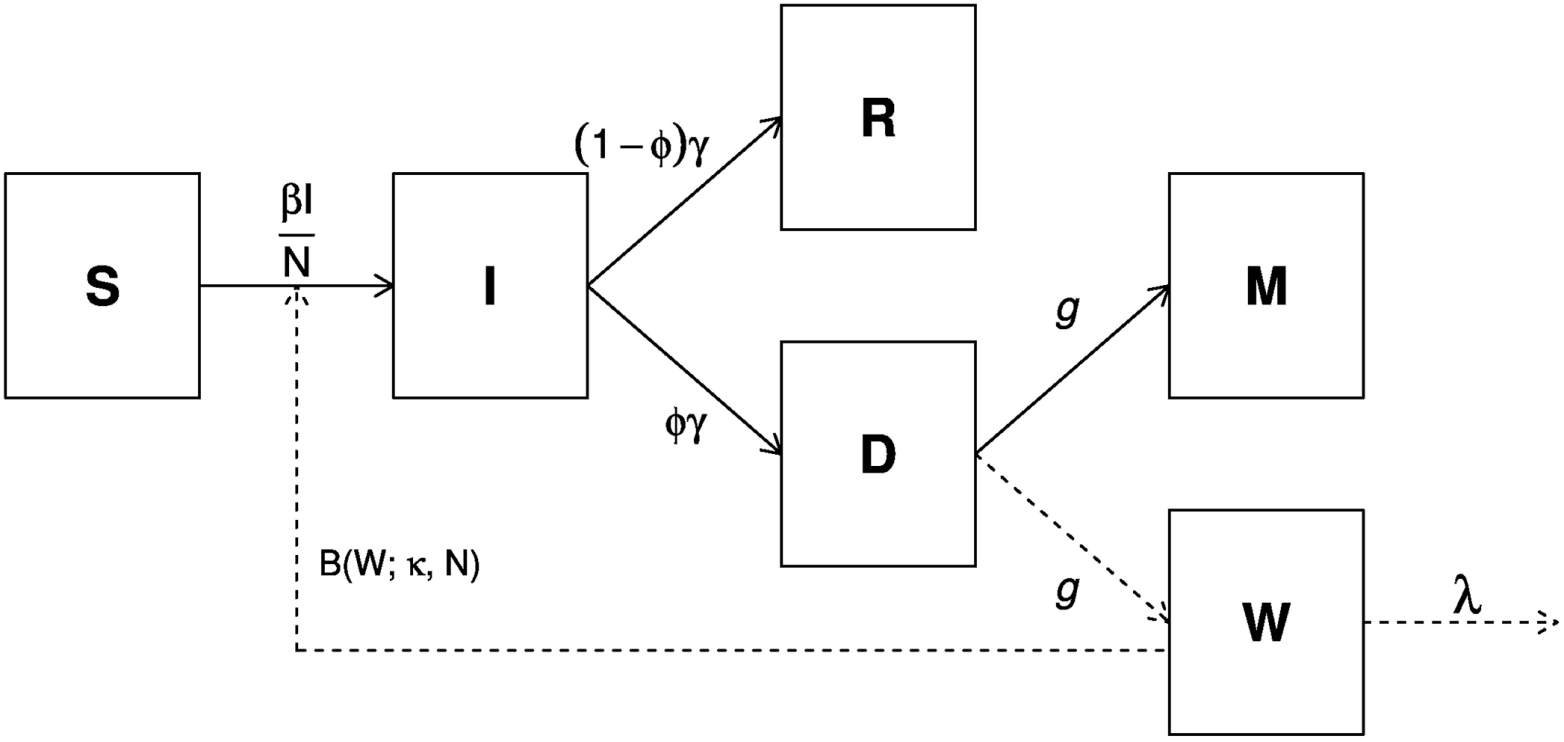
Schematic diagram showing the transmission dynamics during an influenza pandemic. *S*, *I* and *R* denote the number of susceptible, infectious, and recovered individuals, respectively; *D* denotes the number of infected individuals who are no longer infectious and are progressing to death in influenza or pneumonia causes; *M* denotes the cumulative number of influenza-related deaths; and *W* denotes recent influenza mortality, a proxy indicator for the perception of pandemic severity.

We fit the model as described in Fig 2 to the reported weekly influenza deaths from the three largest cities: London boroughs, Birmingham and Liverpool during the 1918-1919 influenza pandemic. We further modelled for all 334 administrative units in England and Wales by incorporating their respective population sizes (*N*), and using the epidemiological parameters of the three cities. A key difference from previous models by Bootsma and Ferguson [8] and He et al. [6] is that they used distinct parameters for different administrative units. By using a common set of parameters, we greatly reduced the number of free parameters and computational time in our work. Previous studies showed that the transmissibility during the pandemic showed little spatial variations [13, 14], thus our assumption is also biologically plausible.

We simulated the epidemic dynamics in Eq (2) using the Partially Observed Markov Process model (POMP, also known as Hidden Markov Model) within a plug-and-play framework [15]. Using the iterated filtering method [16, 17], we computed the maximum likelihood estimate of the following parameters: baseline transmission rate (*β*_0_), case-fatality ratio (*ϕ*), impact of school term (*α*), impact of air temperature (*ξ*), intensity of reactive social distancing (*κ*), decay rate of reactive social distancing (*λ*), and school term start date (*t*_1_) and end date (*t*_2_). We used Euler-multinomial algorithm with a fixed daily time step and incorporated the daily temperature and school dates for model-fitting. We accounted for measurement noise and used negative binomial process to compute the maximum log-likelihood (See S1 Text for details). The POMP model has been widely used in infectious diseases modelling studies, including Ebola, cholera, malaria, influenza, as well as studies in finance and ecological dynamics [15, 18–35]. The POMP package in R was implemented (http://kingaa.github.io/pomp/).

We performed model fittings of the POMP package on workstations. We transformed all parameters into the range (−∞, ∞), by taking logarithm on all positive parameters, or using the logistic function to the parameters that have values between 0 and 1. We used the Iterated Filtering method for multiple times to achieve convergence of model fitting. At each iteration, the current estimates were used as the initial parameter values to ensure that the log-likelihood is improved at each iteration. In order to check that the maximum log-likelihood is indeed a true global maximum, we chose to fix each parameter at several points (e.g. 20 points) across a wide range of values and perform iterated filtering on other parameters. This step yielded the maximum log likelihood as a function of this parameter. Consequently, we achieved convergence of the maximization on smooth maximization profiles.

The likelihood-based inference framework is further discussed in S1 Text and S1 Fig. Some oscillations induced by reactive social distancing are discussed in S1 Appendix.

## Results

### Baseline fitting results

**Fig 3:**
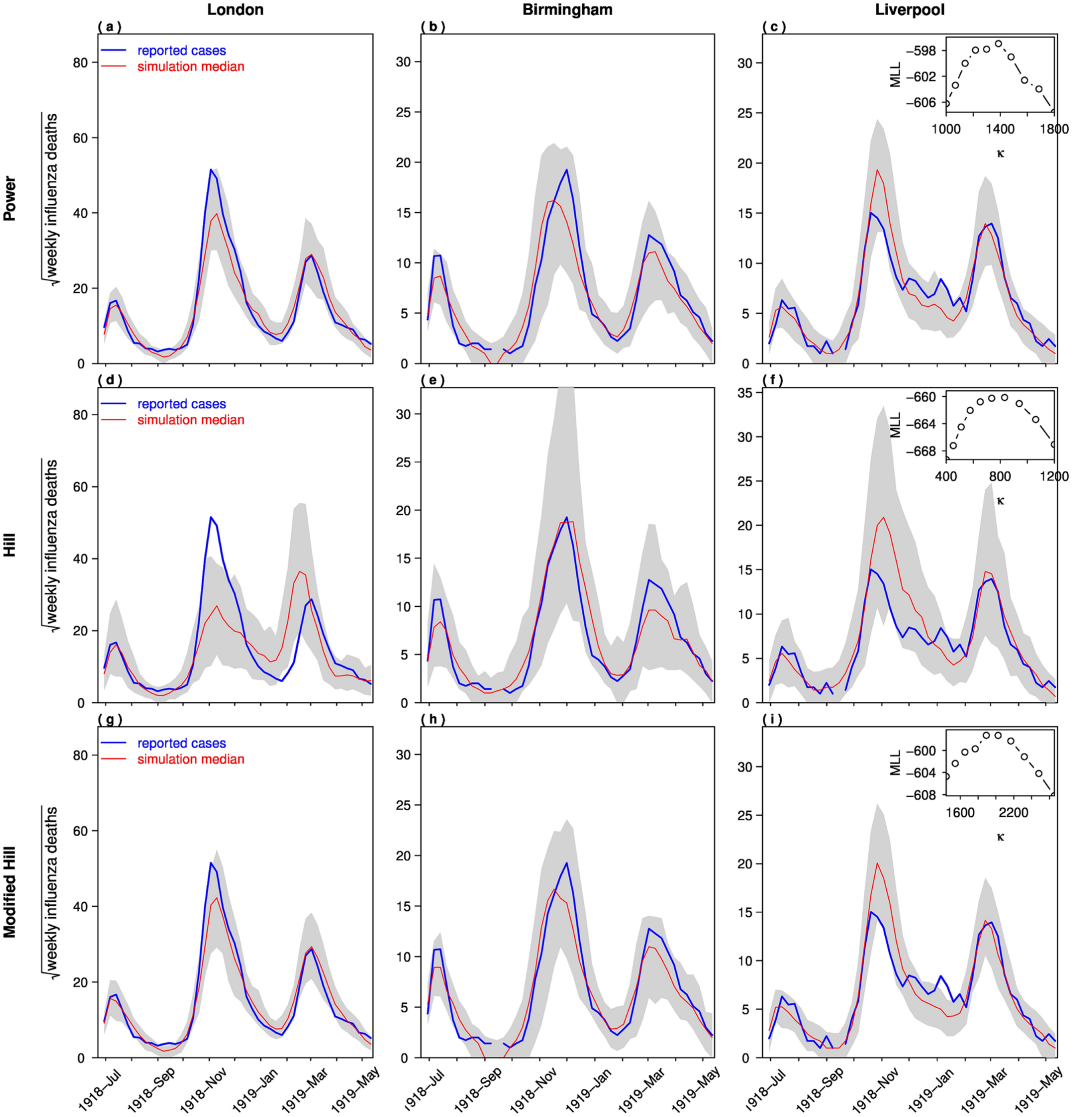
Simulation comparisons of the weekly influenza mortality during the 1918 pandemic in London boroughs, Birmingham and Liverpool. We modelled using three different behavioral functions: (a-c) Power function, (d-f) Hill function, and (g-i) modified-Hill function. Bold blue line: reported cases; thin red line: simulation median. The shaded area indicates the 95% confidence interval. The inset panels show the profile log-likelihood as a function of *κ*.

Fig 3 shows the best-fitting simulation models using three different behavioral functions, i.e. a model with the Power function (Fig 3(a)–3(c)), the Hill function (Fig 3(d)–3(f)) and the modified-Hill function (Fig 3(h)–3(j)). The inset panels show the log-likelihood profile of each model as a function of the parameter *κ*. Since the number of parameters of the three models are the same, their maximum log-likelihoods (MLL) could be directly compared. The MLLs for the Power function, the Hill function and the modified-Hill function are -596.12, -659.58 and -596.34, respectively. Since a larger MLL indicates a better model fit, and the Power function and the modified-Hill function have very similar goodness-of-fit levels, we concluded that these two functions provided the best model choice in this study.

Previous studies assumed key parameters such as $R_{0}$ and *κ* to be different for each city in order to achieve the best model fit [6, 8]. In contrast, our model uses a common set of parameters (i.e. same *κ* and $R_{0}$ in the three cities) but different population sizes and initial conditions for each of the three cities. Of these models, the Power function and the modified-Hill function demonstrated good model fit (Fig 3).

**Fig 4:**
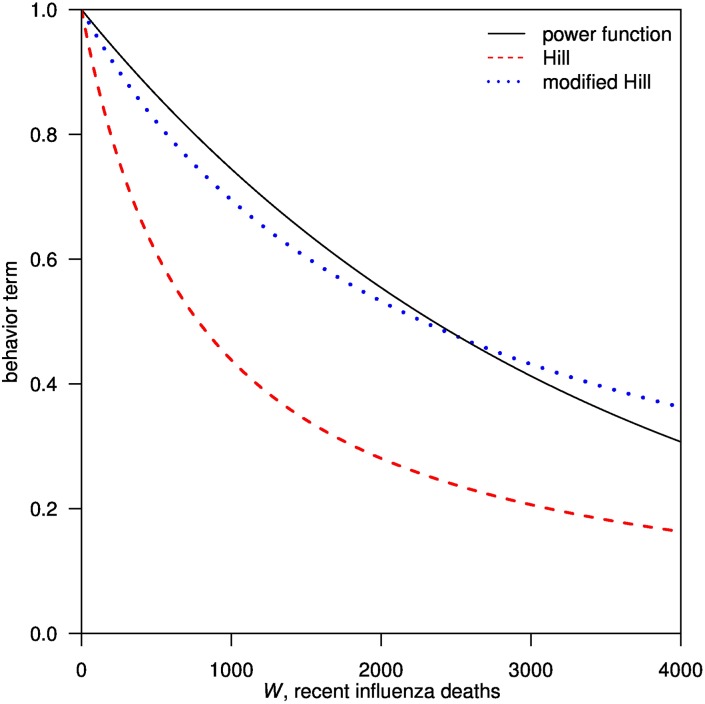
Comparison among the values of three behavioral functions with their best-fitted *κ*.

Fig 4 compares the three behavioral functions with the maximum likelihood estimates of *κ*. The Power function and the modified-Hill function largely overlaps, but the Hill function clearly deviates from the other two functions.

**Fig 5:**
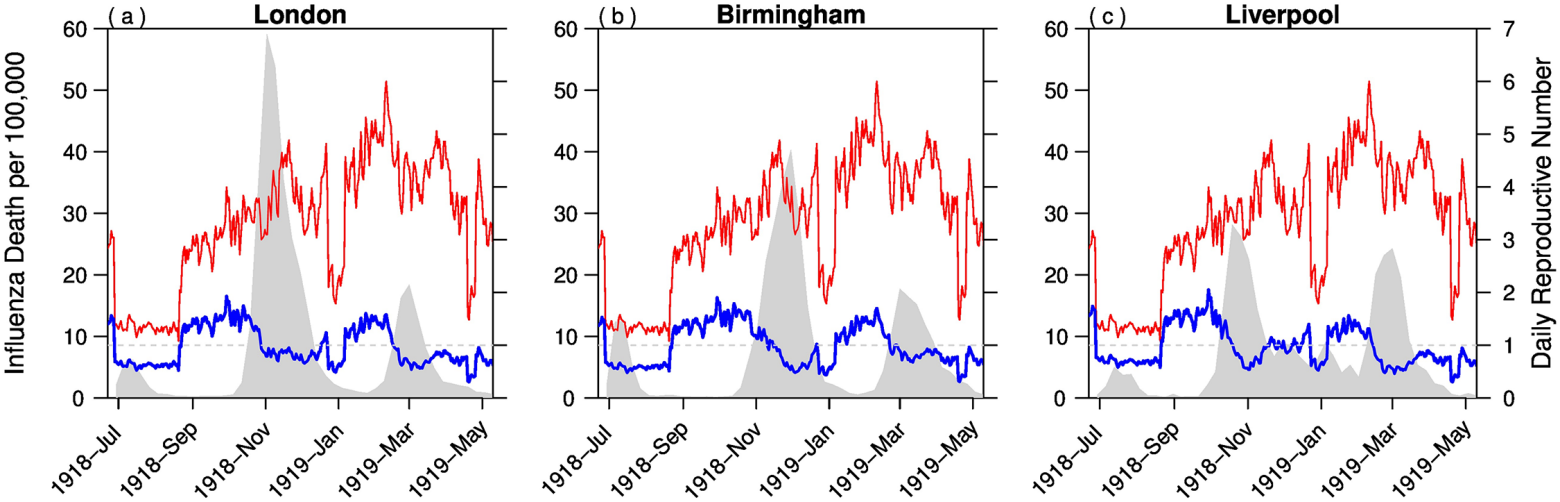
Estimated daily basic reproductive number $R_{0}$ (thin red curve), effective reproductive number $R_{\text{eff}}$ (bold blue curve) and weekly influenza mortality (shaded region). The average basic reproductive number is 3.24 in the three cities.

Fig 5 shows the estimated daily reproductive number *R*_0_ (thin red curve) and the effective reproductive number, $R_{\text{eff}}$ (blue bold curve). Since we assumed all input parameters to be identical, the estimated daily reproductive numbers were identical in all three cities. We set *W* = 0, therefore fluctuations in daily reproductive numbers can only be due to changes in school terms and daily temperature. For the effective reproductive number, we used the estimated *W*(*t*) and the susceptibles, *S*(*t*). Thus $R_{\text{eff}}$ is different between the cities. When $R_{\text{eff}}$ is different from 1, the mortality curve changes with a time lag of about 12 days.

We summarised all parameters estimated in the best-fit model that uses a Power function in Table 1. All parameter values are largely biologically reasonable [6, 8]. Thus, we found a model with common parameter values in all three cities. The only differences are in the population sizes and the initial conditions, where we denoted the initial susceptible population and initial population size to be *S*_0_ and *I*_0_, respectively. The estimated initial conditions were similar among the three cities.

**Table 1:** Summary of all parameters estimated in the best-fit model using the Power function. Distinct parameters could have different values for the three cities. Common parameters have the same values for all three cities.

Parameter	London boroughs	Birmingham	Liverpool	Type
initial, *S*_0_/*N*	0.685 (0.503, 0.836)	0.677 (0.450, 0.806)	0.632 (0.253, 0.950)	distinct
initial, *I*_0_	9552 (7116, 14611)	3760 (2302, 4964)	927 (610, 1521)	distinct
behavioral, *κ*		1323.2 (1185.9, 1484.8)		common
delay, *λ*^−1^ (days)		12.43 (10.73, 14.55)		common
CFR, *ϕ*		0.0118 (0.0108, 0.0129)		common
baseline, *β*_0_		4153.8 (3067.9, 8424.1)		common
school-term, *α*		0.437 (0.377, 0.498)		common
temperature, *ξ*		0.04048 (0.03441, 0.04568)		common
summer vacation start, *t*_1_		June 23 (May 24, June 28)		common
summer vacation end, *t*_2_		August 21 (Aug 12, August 31)		common

### Impact of reactive social distancing

As the goodness-of-fit of the models were similar between the Power function and the modified-Hill function, we chose the Power function for further exploration of the theoretical impacts of reactive social distancing on the pandemic. The theoretical impact depends on the intensity parameter (*κ*) and the decay parameter (*λ*). We introduced the final epidemic size (*Z*) and the weekly mortality which reflect the magnitude of the pandemic.

The basic reproductive number $R_{0}$ is the key epidemiological parameter in infectious disease transmission. It is defined as the average number of secondary cases arising from an average primary case in an entirely susceptible population [36]. If $R_{0}>1$, the disease will spread. However, if *R*_0_ < 1, the disease will not spread [37].

The final epidemic size is the proportion of a population who have been infected during an epidemic. It is determined when the epidemic ends and there are no more infectious individuals in the population. Suppose *s*(∞) is the proportion of susceptible at the end of the epidemic. Then the final size of the epidemic can be defined as 1 − *s*(∞). Without human behavioral intervention, *s*(∞) is the root of the final-size equation [38, 39],

$$\begin{array}{c} \ln s(\infty )=R_{0}\left(s\left(\infty \right)-1\right). \end{array}$$ (4)

Therefore, the final epidemic size can be calculated as follows:

$$\begin{array}{c} Z=1-e^{-R_{0}Z}. \end{array}$$ (5)

**Fig 6:**
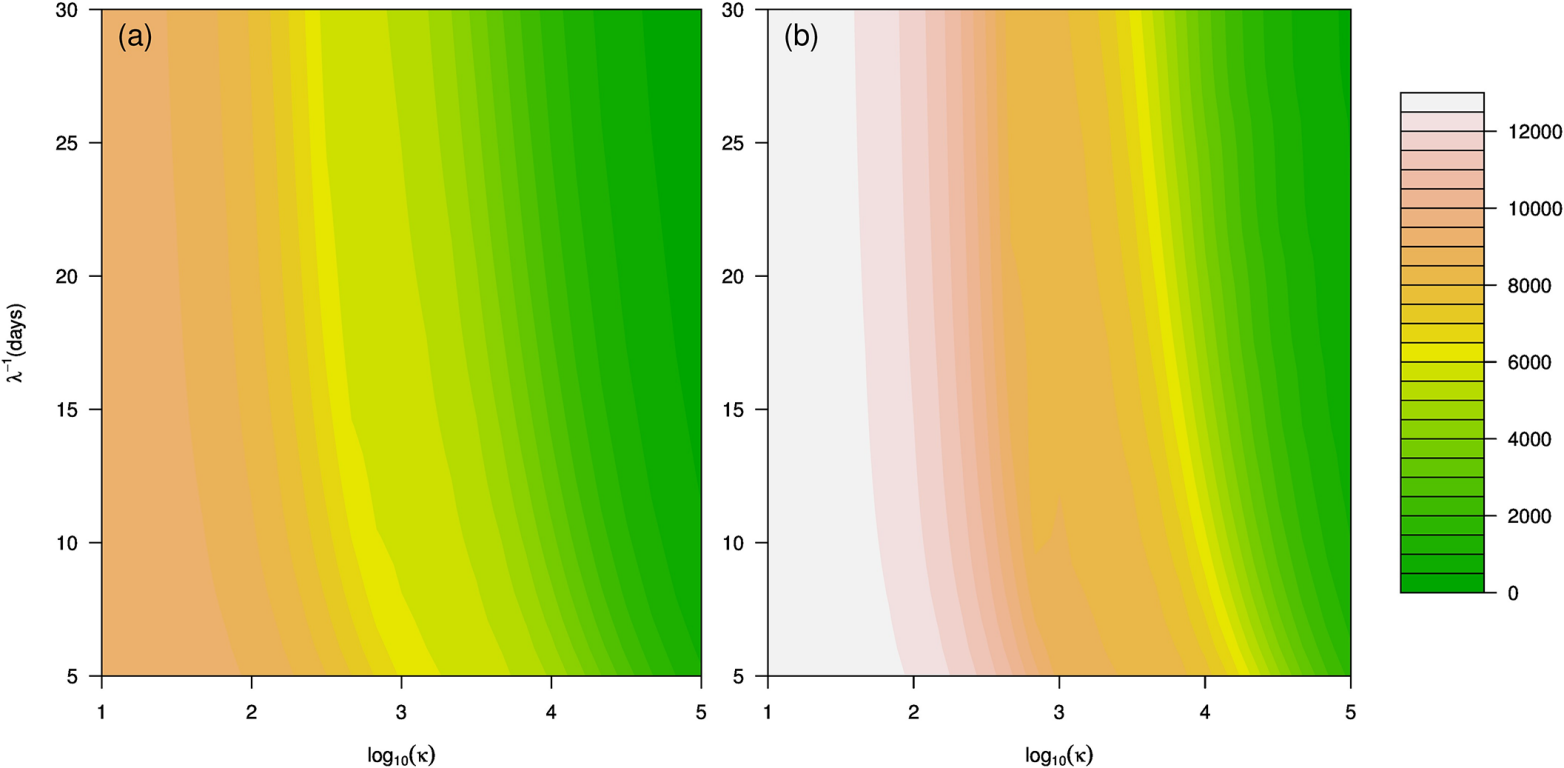
Contour plots of the cumulative number of deaths with $R_{0}=1.5$ (panel a) and $R_{0}=2$ (panel b). *N* = 2,000,000, *S*_0_ = 0.8*N*, *I*_0_ = 100, *g*^−1^ = 8, *γ*^−1^ = 4, *ϕ* = 0.01. *κ* represents the intensity of reactive social distancing behavior, and *λ* represents the rate of decay of reactive social distancing behavior.

**Fig 7:**
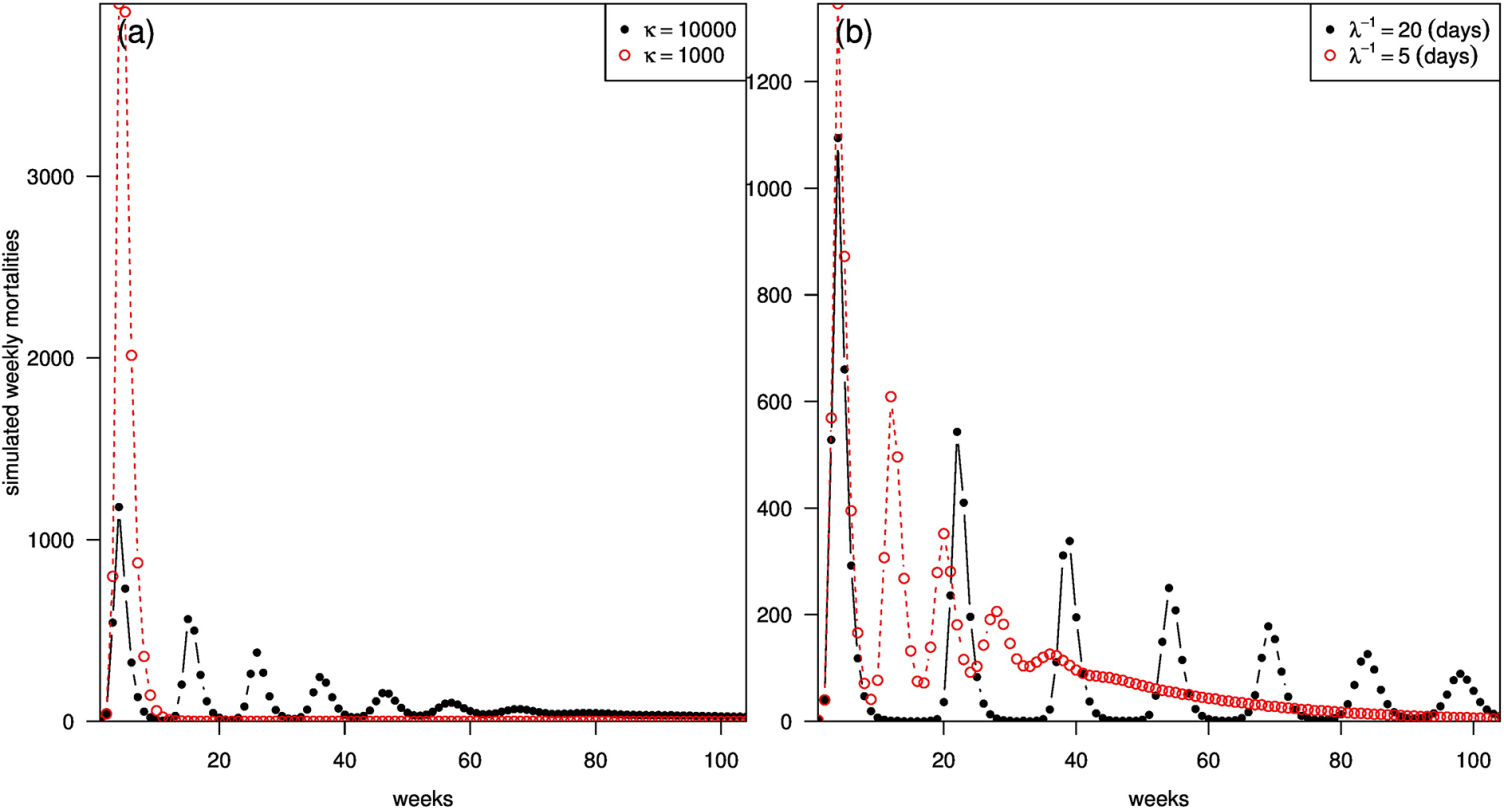
Cumulative number of weekly mortality using different values of *κ* and *λ*. With *N* = 2,000,000, *S*_0_ = 0.8*N*, *I*_0_ = 100, *g*^−1^ = 8, *γ*^−1^ = 4 and *ϕ* = 0.01, the effects of *κ* and *λ* on the simulated weekly mortalities are shown in panels (a) and (b) respectively. In panels (a), we fixed *λ*^−1^ = 10 days, and the cumulative weekly mortalities are 16% smaller when we have *κ* = 10,000 than *κ* = 1,000. In panel (b), when we fixed *κ* = 10,000, the cumulative weekly mortalities will be 27% smaller when we have *λ*^−1^ = 5 days than *λ*^−1^ = 20 days.

With the inclusion of the behavioral term, the effective reproductive number is smaller, which leads to a smaller final epidemic size. However, a closed-form solution could not be obtained, and numerical simulation is needed. Figs 6 and 7 show the simulation results of the impact of the behavioral term on the final epidemic size and weekly mortality, respectively.

Fig 7 shows the cumulative number of weekly mortality by varying the values of the intensity parameter (*κ*) and decay parameter (*λ*) in the behavioral term. In Fig 7(a), increasing *κ* from 1,000 to 10,000 generates oscillations in the simulated weekly mortalities. In Fig 7(b), by fixing *κ* at 10,000, *λ* serves as a tuning parameter which changes the frequency and duration of the oscillations.

### Applying the model to 334 administrative units

**Fig 8:**
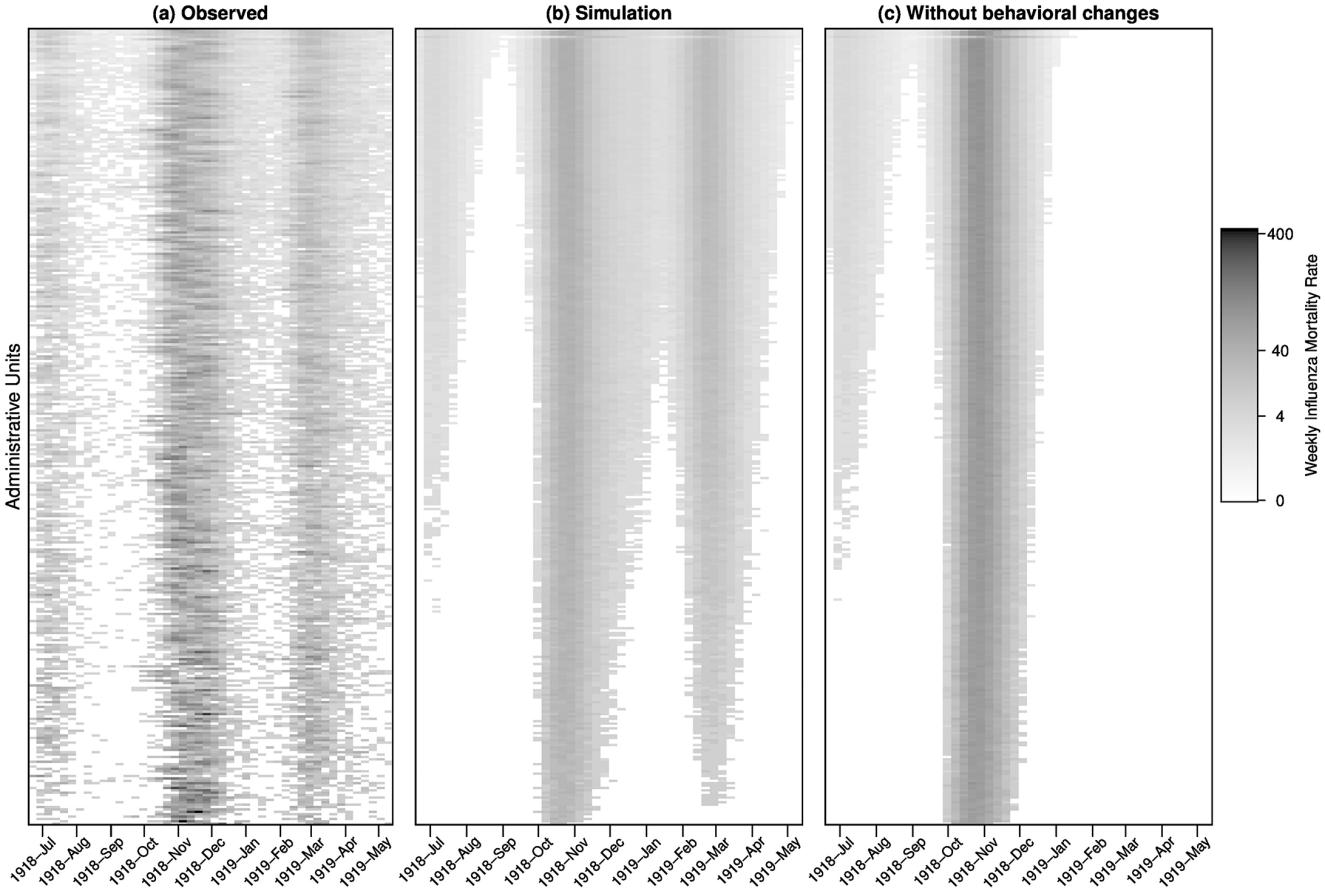
Comparison between the observed and simulated patterns of influenza mortality in 334 administrative units. (a) Observed data. (b) Simulated data that considers school term, temperature, and behavioral changes. (c) Without behavioral changes. Administrative units are ordered in descending population sizes from top to bottom.

We applied our model with parameter values from fitting the three largest cities to all 334 administrative units in England and Wales. The only parameter we need to incorporate into this model are the population sizes of the administrative units. As the three cities had similar initial conditions, we used that of Liverpool’s. The results are displayed in Fig 8. We computed the Pearson’s correlations between the observed (Fig 8(a)) and simulated data that considers school term, temperature and behavioral changes (Fig 8(b)) for each administrative unit. The median correlation is 0.636. This shows that our model performs reasonably well in at least half of the 334 administrative units using only data from three major cities. Previous studies showed that the model fit is inadequate when the behavioral terms are removed, even if the cities are being fitted separately [6, 8]. In Fig 8(c), we set *W* = 0 and using other parameters from Fig 8(b), we show the simulation results of the 334 administrative units. The model can only yield the first two waves with the third wave missed. Furthermore, we compared the overall attack rates in the two scenarios: using all three factors, the estimated infection attack rate is about 28.5% (95% confidence interval (CI): 14.1%, 35.9%). Without behavioral changes, the estimated infection attack rate is about 40.8% (95% CI: 34.0%, 46.9%). Thus, there are substantial reduction in attack rates due to behavioral changes.

We discussed the oscillations induced by reactive social distancing in S1 Appendix. We obtained an interesting linear relationship between the durations of behavioral reaction and the period of transient oscillations (S1 Appendix).

## Discussion and conclusions

Our study compared three forms of behavioral functions that described reactive social distancing, which assumes individuals either respond to the number of recent mortality or the proportion of mortality in the overall population. Using simple epidemic models to fit mortality cases in London boroughs, Birmingham and Liverpool, we showed the behavioral functions in the form of the Power function or the modified-Hill function outperformed the Hill function in terms of model fit. These results suggest that the proportion of mortality plays a more important role than the number of recent mortality.

Our modelling techniques are an improvement compared with earlier studies [6, 8]. We showed that a common set of parameters and temperature from Central England could be used for modelling the 334 administrative units. Bootsma and Ferguson [8] developed an epidemic model to study the impacts of public health interventions on the 1918 influenza pandemic in 16 US cities. He et al [6] proposed another epidemic model which incorporates school opening and closing, temperature changes and changes in human behavioral responses during the 1918 influenza pandemic in 334 administrative units in England and Wales. However, in both studies, instead of using a common set of model input parameters, unique model input parameters were needed for model fitting of each city or administrative units. Here, our model requires only a common set of parameters for the three-city or the subsequent 334-administrative unit model-fitting procedure, and the reduced number of parameters used represented significant improvement in computational efficiency and resulted in more robust estimates. Caley et al. [40] studied the 1918 influenza pandemic in Australia and showed that reactive social distancing had a significant impact on the observed multiple epidemic wave and final epidemic size. Our effective reproductive numbers are comparable to these studies.

Our theoretical damping oscillation results provide a plausible explanation to the observed multiple waves, where reactive social distancing in response to the high recent proportion of influenza deaths could lead to a dampening of epidemic waves. However, with the decline in the proportion of influenza deaths, public risk perception could be lowered as well, leading to less social distancing which could eventually induce another epidemic wave. In addition, we showed that reactive social distancing could lead to reduction in final epidemic size.

Our findings are plausible and are consistent with earlier mathematical modelling studies on the 1918 influenza pandemic. Our estimated initial proportion of susceptible individuals are around 0.650, which are comparable with previous studies by Mathews et al [41], Bolton et al. [42], Gani et al. [43] and He et al. [6]. Our estimates of the initial number of infectious individuals and school term intensity are also consistent with He et al [6].

Compared to previous studies, our methods provide several improvements. First, by using a common set of model parameters for fitting the three-city model, we have greatly enhanced our computational efficiencies and have also resulted in more robust estimates of the final epidemic size. Second, by comparing different forms of social distancing, we identified that people responded most to the proportion of influenza mortality during the 1918 pandemic. Third, our theoretical results suggest an almost perfect linear relationship between the mean period of damping oscillations and the duration of reactive social distancing under ideal conditions. These findings have important implications on the impact of behavioral reaction on influenza pandemic waves.

Major limitations of our study include the lack of direct historical behavioral data on quantifying the extent of reactive social distancing. Also, other non-pharmaceutical interventions could have played a role on the influenza pandemic patterns observed, but these measures are not considered in our model. There could be differences in summer vacation periods and daily temperature data in the three cities. However, such detailed data are not accessible to us. In future epidemics or pandemics, such information can be gathered and incorporated into the framework developed in this work. Furthermore, our model failed to achieve reasonable model fits for small administrative units due to their smaller population sizes because the model parameters are estimated from large cities. We were indeed more interested in fitting large cities. Finally, we did not consider the effect where epidemics in small administrative units are likely driven by large cities.

In conclusion, a simple model that considers reactive social distancing, temperature, and school term could explain the observed multiple waves and final epidemic size in London boroughs, Birmingham and Liverpool during the 1918 influenza pandemic. Despite societal changes, our historical analyses on the 1918 pandemic could still serve as an evidence base for future pandemic planning.

## Supporting information

S1 Text Likelihood based inference framework. (PDF)

S1 Fig Temperature in England and Wales 1918-1919. Temperature data in Central England from June 29, 1918 to May 10, 1919 was downloaded from the UK Met Office Hadley Centre for Climate Change.
(PDF)

S1 Appendix Oscillations induced by reactive social distancing. (PDF)
