# Supplementary material for: Effects of reactive social distancing on the 1918 influenza pandemic
Source: PLoS One. 2017 Jul 12;12(7):e0180545. doi: 10.1371/journal.pone.0180545 (PMC5507503; doi:10.1371/journal.pone.0180545)
Supplement: S1 Text — (PDF) [file pone.0180545.s001.pdf]

S1 Likelihood-based inference framework

The weekly reported mortality cases are obtained as

$$Z_i = \int_{t_i}^{t_{i+1}} \phi \gamma_{h1} I_{h1} dt \quad (1)$$

where ϕ denotes the case-fatality ratio. We assume that the weekly observed mortality cases C_i is a random sample from a Negative-binomial (NB) distribution

$$C_i \sim \text{NB}\left(n = \frac{1}{\tau}, p = \frac{1}{1 + Z_i \tau}\right) \quad (2)$$

where n and p denote the size and probability of the NB distribution, and τ denotes an over-dispersion parameter which will be estimated.

The following likelihood function is for one administrative unit

$$L(\theta_1 | C_{1,\ldots,N}) = \prod_{i=1}^{N} l_i \quad (3)$$

where θ_1 denotes the parameter vector, l_i is the weekly density associated with C_i and Z_i, and N denotes the number of weeks. This function is then applied to three cities: London boroughs, Birmingham and Liverpool, and the overall likelihood is

$$L_{\text{overall}} = \prod_{j=1}^{3} L_j \quad (4)$$

We use iterated filtering based plug-and-play inference framework [1–7] to estimate θ_1 via maximizing L_{overall}.

References

[1] He D, Ionides EL, King AA. Plug-and-play inference for disease dynamics: measles in large and small populations as a case study. J R Soc Interface. 2010; 7:271-83.

[2] Ionides EL, Bretó C, King AA. Inference for nonlinear dynamical systems. Proc Natl Acad Sci U S A. 2006; 103:18438-43.

[3] Ionides EL, Bhadra A, King AA. Iterated Filtering. Ann Stat. 2011; 39:1776-802.

[4] He D, Dushoff J, Day T, Ma J, Earn DJD. Mechanistic modelling of the three waves of the 1918 influenza pandemic. Theor. Ecol. 2011; 4:283-8.

[5] Camacho A, Ballesteros S, Graham AL, Carrat F, Ratmann O, Cazelles B. Explaining rapid reinfections in multiple-wave influenza outbreaks: Tristan da Cunha 1971 epidemic as a case study. Proc Biol Sci. 2011; 278:3635-43.

[6] Earn DJ, He D, Loeb MB, Fonseca K, Lee BE, Dushoff J. Effects of school closure on incidence of pandemic influenza in Alberta, Canada. Ann Intern Med. 2012; 156:173-81.

[7] King AA, Ionides EL, Pascual M, Bouma MJ. Inapparent infections and cholera dynamics. Nature. 2008; 454:877-80.
