# Supplementary figures and images for: Effects of reactive social distancing on the 1918 influenza pandemic
Source: PLoS One. 2017 Jul 12;12(7):e0180545. doi: 10.1371/journal.pone.0180545 (PMC5507503; doi:10.1371/journal.pone.0180545)

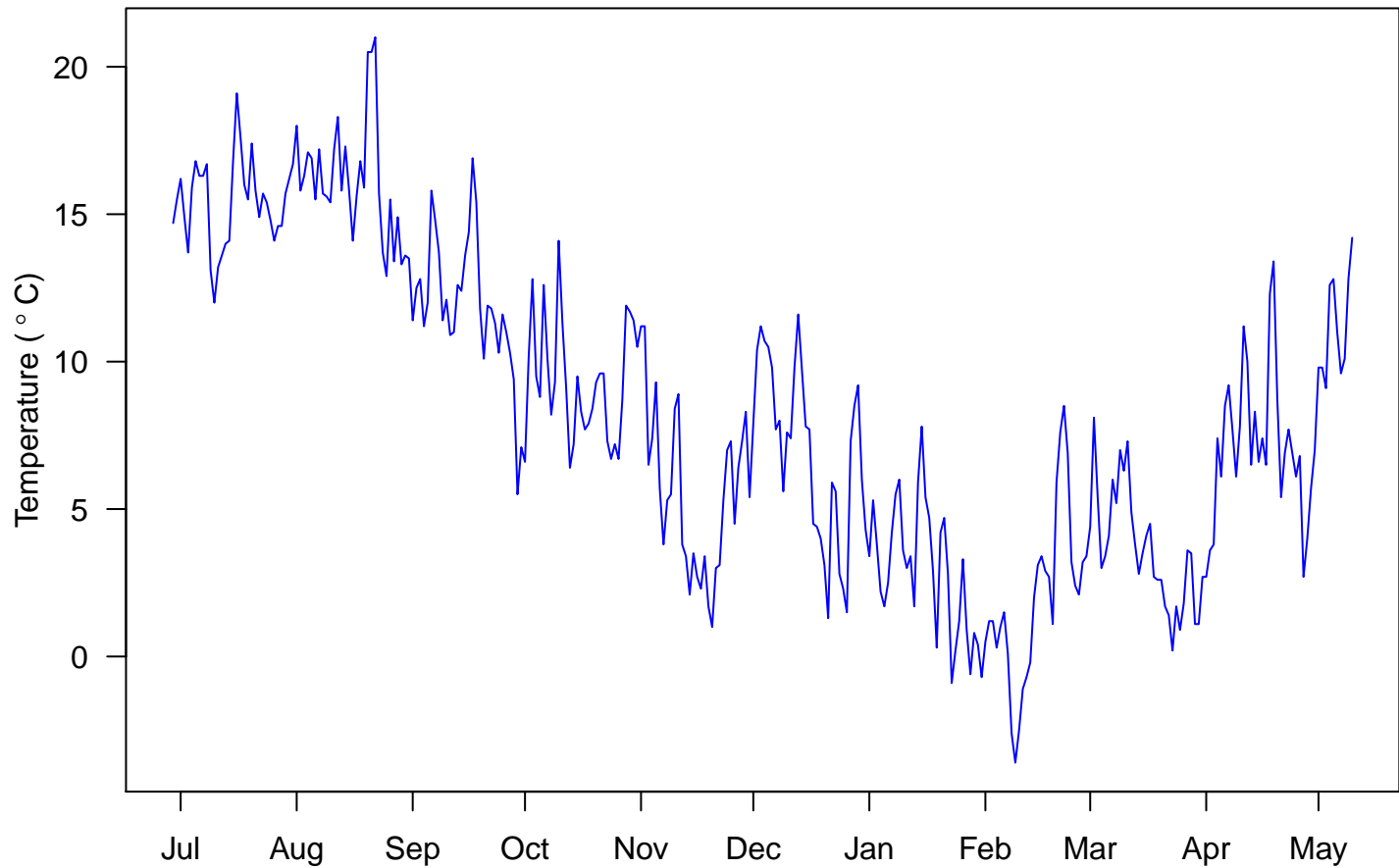

Supplement: S1 Fig — Temperature data in Central England from June 29, 1918 to May 10, 1919 was downloaded from the UK Met Office Hadley Centre for Climate Change. (PDF) [file pone.0180545.s002.pdf]
