# Supplementary material for: Effects of reactive social distancing on the 1918 influenza pandemic
Source: PLoS One. 2017 Jul 12;12(7):e0180545. doi: 10.1371/journal.pone.0180545 (PMC5507503; doi:10.1371/journal.pone.0180545)
Supplement: S1 Appendix — (PDF) [file pone.0180545.s003.pdf]

S3 Oscillations Induced by Reactive Social Distancing

If we only consider the impact of reactive social distancing on transmission rate, then $\beta = \beta_0[1 - \frac{W(t)}{N}]^\kappa$. We further assume that the size of the susceptible population is constant. A simplified model can be re-written as:

$$\begin{aligned} \dot{I} &= \beta_0(1 - \frac{W}{N})^\kappa \frac{SI}{N} - \gamma I, \\ \dot{D} &= \gamma\phi I - gD, \\ \dot{W} &= gD - \lambda W. \end{aligned} \tag{1}$$

Applying the standard formula for reproductive numbers, we obtain $\mathcal{R}_0 = \beta_0 S/(\gamma N)$. We denote the disease free equilibrium (DFE) as $E_0 = (0, 0, 0)$. If $\mathcal{R}_0 < 1$, the DFE is globally and asymptotically stable. On the other hand, if $\mathcal{R}_0 > 1$, there is an endemic equilibrium $E^* = (I^*, D^*, W^*)$, where

$$I^* = \frac{\lambda W^*}{\gamma\phi}, \quad D^* = \frac{\lambda W^*}{g}, \quad W^* = N(1 - \mathcal{R}_0^{-\frac{1}{\kappa}}). \tag{2}$$

In order to analyze the stability of an endemic equilibrium, we obtain the Jacobian matrix of the model (1) at E^* as

$$J(E^*) = \begin{pmatrix} 0 & 0 & -\frac{\lambda\kappa}{\phi}(\mathcal{R}_0^{\frac{1}{\kappa}} - 1) \\ \gamma\phi & -g & 0 \\ 0 & g & -\lambda \end{pmatrix}. \tag{3}$$

Thus the characteristic function is

$$f(x) = x^3 + (g + \lambda)x^2 + g\lambda x + g\lambda\kappa\gamma(\mathcal{R}_0^{\frac{1}{\kappa}} - 1). \tag{4}$$

Fig 7 shows the damping oscillation. Since we are more interested in the periodic solution of the model (1), we aim to find a condition for the existence of a pair of purely imaginary roots of equation $f(x) = 0$. Let $x_{1,2,3}$ be the three eigenvalues, using Vieta's formulas we have

$$\begin{cases} x_1 + x_2 + x_3 = -(g + \lambda), \\ x_1x_2 + x_1x_3 + x_2x_3 = g\lambda, \\ x_1x_2x_3 = -g\lambda\kappa\gamma(\mathcal{R}_0^{\frac{1}{\kappa}} - 1). \end{cases} \tag{5}$$

If there exists a pair of imaginary roots of equation $f(x) = 0$, then

$$G(\beta_0, \gamma) = g + \lambda - \kappa\gamma(\mathcal{R}_0^{\frac{1}{\kappa}} - 1) = 0. \tag{6}$$

The real eigenvalue is $x_1 = -(g + \lambda) < 0$. The other two purely imaginary eigenvalues are:

$$x_{2,3} = \pm i\sqrt{g\lambda}. \tag{7}$$

If we model the reactive social distancing term as $e^{-\kappa\frac{W(t)}{N}}$, we can result in the same eigenvalues as we have in the Power function. The condition of the existence of purely imaginary eigenvalues is different from (6), which is

$$G^{'}(\beta_0, \gamma) = g + \lambda - \gamma\ln(\mathcal{R}_0). \tag{8}$$

But when $\kappa \gg 1$, $ln(\mathcal{R}_0)$ and $\kappa(\mathcal{R}_0^{\frac{1}{\kappa}} - 1)$ will have the same linear terms as in their Taylor expansion at $\mathcal{R}_0 = 1$.

Theorem 0.1 *If $\gamma N/S < \beta_0 < \gamma N/S((g + \lambda + \gamma\kappa)/(\gamma\kappa))^\kappa$, the endemic equilibrium is locally and asymptotically stable; If $\beta_0 > \gamma N/S((g + \lambda + \gamma\kappa)/(\gamma\kappa))^\kappa$, the endemic equilibrium is unstable. When $G(\beta_0, \gamma) = 0$, a Hopf bifurcation occurs. Model (1) has a stable periodic solution.*

The proof is similar to Liu et al. [1] and is omitted here. Based on the above analyses, when the change of S is relatively slow, the oscillation phenomenon can theoretically occur in our model, as we have observed both in our simulations (e.g. Fig 7) and in reality. However, the size of the susceptible population will become smaller over time rather than staying constant, and this leads to damping oscillations of weekly infections (and mortality). In Fig 1, we focused on the first two years in a typical population setting ($N = 2,000,000$, $\kappa = 100000$, $S_0 = 0.8N$, $I_0 = 100$), and we showed a perfectly linear relationship between the mean period of the damping oscillation and the duration of social distancing (λ^{-1}). Such relationships hold for a variety of combinations of γ^{-1} and g^{-1}. These linear relationships cannot be explained by the endemic equilibrium analysis. Fig 2 shows the impact of the κ and λ on the frequency and period of the damping waves. From panel (b), we can see that in the upper range of κ, there is a region of linear relationship between the period and λ^{-1}. Further work is needed to determine the analytical formula for this.

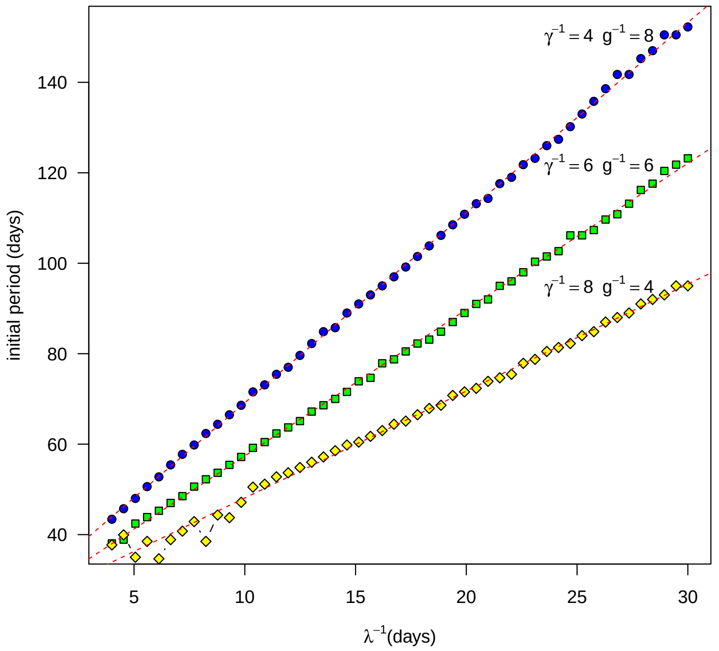

Figure 1: With $N = 2,000,000$, $\kappa = 100,000$, $\phi = 0.01$, $S_0 = 0.8N$, $I_0 = 100$. The linear relationship between the duration of social distancing (λ^{-1}) and the mean period of damping oscillations (in days) with three different combinations of γ^{-1} and g^{-1}. We have $\gamma^{-1} = 8$ and $g^{-1} = 4$ (depicted in yellow diamonds), $\gamma^{-1} = 6$ and $g^{-1} = 6$ (depicted in green squares) and $\gamma^{-1} = 4$ and $g^{-1} = 8$ (depicted in blue circles), we noted an almost perfectly linear relationship using these parameter combinations.

References

[1] Liu R, Wu J, Zhu H. Media/psychological impact on multiple outbreaks of emerging infectious diseases. Comput Math Methods Med. 2007; 8: 153–64.

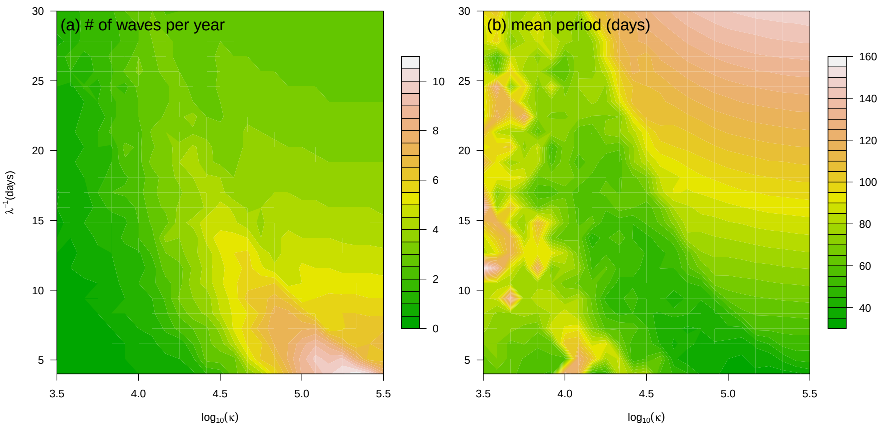

Figure 2: Impacts of κ and λ on the wave dynamics. Panel (a) shows the number of waves per year and panel (b) shows the mean period of waves in days. We conducted simulations of wave dynamics for the first ten years, with parameters fixed at $N = 2,000,000$, $\gamma^{-1} = 4$ days, $g^{-1} = 8$ days, $\phi = 0.01$, $S_0 = 0.8N$, and $I_0 = 100$.
